# Influence of Orthodontic Treatment on Changes in the Maxillary Sinus Dimensions

**DOI:** 10.7759/cureus.53363

**Published:** 2024-02-01

**Authors:** Eiji Tanaka, Hiroshi Yamada, Masaaki Higashino, Masaki Sawada, Saya Suetake, Susumu Abe

**Affiliations:** 1 Orthodontics and Dentofacial Orthopedics, Tokushima University Graduate School of Biomedical Sciences, Tokushima, JPN; 2 Orthodontics, Yamada Orthodontic Office, Izumiotsu, JPN; 3 Otolaryngology, Osaka Medical and Pharmaceutical University, Osaka, JPN; 4 Comprehensive Dentistry, Tokushima University Graduate School of Biomedical Sciences, Tokushima, JPN

**Keywords:** volumetric change, orthodontic treatment, maxillary sinus, craniofacial morphology, computed tomography

## Abstract

Objective

This study aimed to investigate the correlation of craniofacial morphology with maxillary sinus morphology and to evaluate whether orthodontic treatment facilitates maxillary sinus enlargement in adults.

Materials and methods

A total of 45 adult women underwent cephalography and computed tomography before and after orthodontic treatment. All participants were classified into three groups: skeletal class I, II, and III. The average dimensions and volume of the maxillary sinus were calculated in each subgroup. Furthermore, multiple regression analysis was used to analyze the correlations of maxillary sinus dimensions with 20 cephalometric variables.

Results

Before treatment, the maxillary sinus width, height, depth, and volume were 32.2 ± 3.9 mm, 39.5 ± 3.8 mm, 38.6 ± 1.8 mm, and 36,179.3 ± 5,454.0 mm^3^ in skeletal class I, 33.9 ± 6.2 mm, 37.3 ± 3.5 mm, 38.6 ± 2.4 mm, and 34,729.8 ± 6,686.6 mm^3^ in skeletal class II, and 32.0 ± 4.3 mm, 41.8 ± 5.0 mm, 38.0 ± 2.8 mm, and 35,592.3 ± 10,334.3 mm^3^ in skeletal class III, respectively. Despite no significant differences in maxillary sinus width, depth, or volume, the height was significantly lower in the skeletal class II than in the other two. Regardless of the skeletal pattern, maxillary sinus height and volume increased considerably after treatment. Moreover, the maxillary sinus width was substantially involved in pretreatment U1 to SN and overbite and posttreatment U1 to NA and overjet.

Conclusion

Except for the height, the maxillary sinus dimensions were almost similar, irrespective of the skeletal classification. The posttreatment sinus height and volume were significantly greater than the pretreatment values, although the sinus width and length showed no significant changes during orthodontic treatment. This implies that orthodontic treatment may facilitate the enlargement of the maxillary sinus even after physical growth.

## Introduction

A large volume of the skull is filled with paranasal sinuses, consisting of the maxillary, frontal, sphenoid, and ethmoidal sinuses, which perform critical functions such as air filtration and providing immune barrier. For the sinuses, the inner surfaces are covered with a thin mucus layer, which ensures maintenance of moisture and health [1-4]. In addition, they are in need of biomechanical stimulation to develop and maintain cranial architecture [5]. Thus, masticatory stimuli may be related to sinus development since mastication mainly contributes to the induction of mechanical stress on the craniofacial bones [6].

The maxillary sinus is a multifunctional bony cavity that forms at a late stage in fetal life. It measures approximately 144 mm^3^ at birth and grows pyramidally into adulthood [7]. The sinus floor enlarges to the posterior alveolar process, pneumatizes the alveolar cavity, and, in some cases, causes the penetration of molar root apices into the sinus [7]. It contributes to facial growth, reduction of skull weight, and absorption of traumatic impacts on cranial skull structures [8]. At 8-12 years of age, the maxillary sinus reaches its full size and stabilizes after the eruption of the maxillary second molar [9,10]. At approximately 20 years of age, pneumatization of the maxillary sinus ceases [11].

During orthodontic movement of the tooth along with the maxillary sinus, the migrating root is moved into the alveolar bone by surrounding bone resorption and apposition. Bone resorption and apposition are balanced in response to the orthodontic force. Alveolar bone modeling and remodeling systems are able to adapt rapidly to changes in mechanical loading [12]. New bone formation on the sinus floor can be stimulated by orthodontic tooth movement [13,14]. Given this information, tooth movement passing through the maxillary sinus has an effect on the sinus dimensions and volume in comparison to the sinus without tooth movement [7,15]. However, predicting the volumetric and dimensional changes in the maxillary sinus after orthodontic treatment is still unclear.

Recently, several studies have suggested that biomechanical stimulation has dimensional and volumetric effects on maxillary sinus in adults [7,10], while other studies have reported less or minimal effects on maxillary sinus dimensions and volume [16,17]. However, the hypothesis remains controversial. Therefore, the aims of this study were to determine the maxillary sinus dimensions and volume in orthodontic adult patients before and after treatment with multibracket appliances using cone-beam computed tomography (CBCT; Alphard-3030, Asahi Roentgen Ind. Co., LTD., Kyoto, Japan) and to identify the association of craniofacial morphology with maxillary sinus morphology. Furthermore, the effect of orthodontic stimulation on maxillary sinus form was elucidated. Considering its location and anatomical variations, the maxillary sinus may reflect posterior occlusion, and changes in the size of the maxillary sinus may be used as an indicator of harmonious posterior occlusion. Taken together, this information may have clinical implications for the prognosis of orthodontic treatment outcomes.

## Materials and methods

Participants

A total of 45 female patients with a variety of malocclusions who underwent orthodontic treatment with multibracket appliances at the Yamada Orthodontic Office from January 2010 to December 2022 were included in this study. All participants gave their informed consent to participate in this study following a thorough explanation of the research objectives and procedures. Patients with a history of otorhinolaryngological diseases, craniofacial trauma, and orthodontic treatment experience were excluded. This study was approved by the Tokushima University Hospital Ethics Committee (permit no. 3900).

In this study, an estimate of the required sample sizes was made. The effect size for the appropriate statistical tests, both parametric and nonparametric, was considered medium (0.25) for the comparison among the three groups described below. G*Power software (Düsseldorf, Germany) was used to calculate statistical power (1-β). Power analysis was performed using the results of a preliminary study for one-way or two-way repeated measures analysis of variance (r-ANOVA) with an effect size of 0.241, a significance level (type I error) of 0.05, and a power level of 0.8 after performing the statistical analysis with a post-hoc test. As a result, the minimum sample size required was calculated to be 15 for each group using a two-way r-ANOVA.

**Figure 1:**
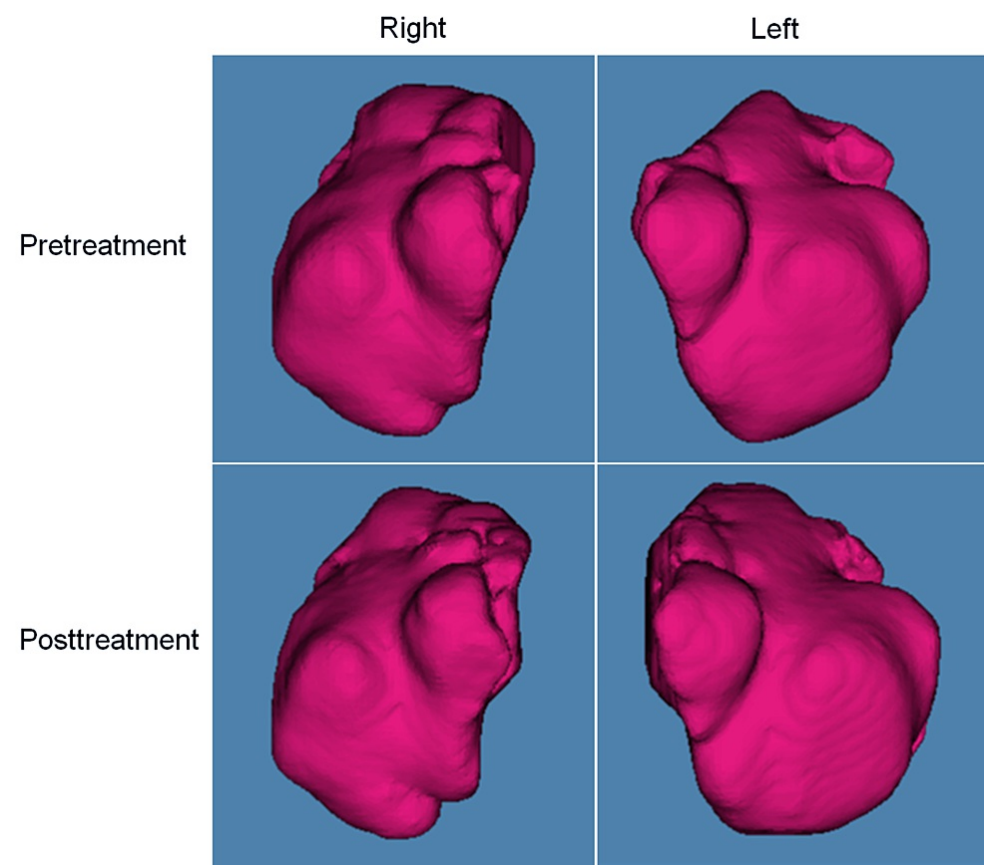
Representative images of the maxillary sinuses.

All patients underwent pretreatment and posttreatment CBCT with the following acquisition parameters: 60-110 kv; 3-15 mA; 0.6-mm collimation, rotation time of 18 seconds; and 0.39-mm reconstruction thickness. A series of images were processed using the Dolphin Imaging System (Dolphin Imaging and Management Solutions, Verona, Italy) throughout orthodontic treatment. A series of CT Digital Imaging and Communications in Medicine (DICOM) images were automatically stacked, and a three-dimensional model of the maxillary sinus and volume-rendering images were extracted (Figure 1). Before and after treatment, lateral cephalograms were also performed using a cephalometric radiographic system (Hyper-X CM; Asahi Roentgen Ind. Co., Ltd.). All lateral cephalograms were obtained at maximum intercuspation. In short, the head of the patient was secured with ear rods, and the forehead pads were stabilized in a position where the Frankfort horizontal plane was parallel to the ground.

A single examiner (H.Y.) traced each lateral cephalogram on acetate paper. Another examiner (M.S.) plotted the tracings on an integrated coordinate system using a digitizer belonging to the Dolphin Imaging System. The precision of the digital plot was ascertained by two orthodontic experts who participated as co-authors in the present study. All examiners were blinded to the participants’ general health state. Prior to the measurements, the intraexaminer reliability of the cephalometric analysis was confirmed using 20 randomly selected cephalograms. The same examiner traced and plotted three arbitrary points (nasion, sella, and pogonion points), twice with an interval of one week, and calculated the intraclass correlation coefficient (ICC). As a result, the ICC was 0.884 (95% CI: 0.713-0.954), indicating acceptable reliability of the selected measures.

Craniofacial morphology

According to the ANB angle indicating the maxillomandibular horizontal jaw-base relationship, the participants were classified into three groups: skeletal class II group (patients with more than 5.0° ANB angle), skeletal class I group (1.0° ≤ ANB angle ≤ 5.0°), and skeletal class III group (less than 1.0° ANB angle). From the lateral cephalography, 11 angular and nine linear measurements were assessed for morphometric evaluation as given below.

Angular measurement items (°)

· SNA: An angle consisting of sella, nasion, and A-point, indicating the anteroposterior position of the maxilla in relation to the anterior cranial base.

· SNB: An angle consisting of sella, nasion, and B-point, indicating the anteroposterior position of the mandible in relation to the anterior cranial base.

· ANB: Angle consisting of A-point, nasion, and B-point, indicating the anteroposterior position of the mandible in relation to the maxilla.

· Gonial angle (Go. A): An angle between the mandibular plane and the mandibular ramus plane.

· FMA: Angle between the mandibular and the Frankfort horizontal planes, indicating the mandibular divergency.

· Occlusal plane to SN (Occl. pl. A): An inclination angle of the occlusal plane to the anterior cranial base.

· Palatal plane to FH (Pal. pl. A): An inclination angle of the palatal plane to the Frankfort horizontal plane.

· U1 to SN: An inclination angle of the maxillary central incisal axis to the sella-nasion line.

· Interincisal angle (IIA): An angle composed of the long axes of the maxillary and mandibular central incisors.

· IMPA: An inclination angle of mandibular central incisal axis to the mandibular plane.

· FMIA: An inclination angle of the mandibular central incisal axis to the Frankfort horizontal plane.

Linear measurement items (mm)

· SN: Anteroposterior length of the anterior cranial base.

· U1 to NA: Length of a perpendicular line from the maxillary central incisal edge to the A-point-nasion line.

· L1 to NB: Length of perpendicular line from the mandibular central incisal edge to the B-point-nasion line.

· Overjet: Horizontal gap of the maxillary and mandibular central incisal edges along the occlusal plane.

· Overbite: Vertical gap of the maxillary and mandibular central incisal edges along the line perpendicular to the occlusal plane.

· N-Me: Anterior facial height between the nasion and the menton.

· Ar-Go: Mandibular ramus height between the articulare and the gonion.

· Ar-Me: Total mandibular length between the articulare and the menton.

· Go-Me: Mandibular body length between the gonion and the menton.

Maxillary sinus morphology

The three-dimensional maxillary sinus models were constructed from CT DICOM data. A specific threshold was set for volumetric measurement of the maxillary sinus. The threshold limits ranged from -100 HU (minimum) to 70 HU (maximum). On each side, the maxillary sinus was identified as an integral part of the air cavity within the sinus walls in the maxillary bone on reformatted axial, sagittal, and coronal images. The maxillary sinus volume was automatically calculated using volume-rendered images. The maximum distance between the most lateral and medial points of each sinus was identified as the width. The height was defined as the maximum distance between the bottom and the highest points of the sinus on each side. The maximum breadth between the most prominent points of the anterior and posterior parts of the sinus was measured as the sinus breadth.

Statistical analysis

Statistical analyses were performed using SPSS software (Version 27.0; IBM Corp., Armonk, NY). The Shapiro-Wilk test was used to examine the normality of each morphometric variable. The mean and standard deviation (SD) of the maximum dimensions and the total volume of the maxillary sinus before orthodontic treatment were calculated for the three skeletal classes according to the ANB angle. First, using one-way ANOVA, the differences in each sinus size and volume before treatment were compared among the three subgroups after the data were evaluated using Levene's test. In addition, the pretreatment and posttreatment differences in sinus dimensions were elucidated and compared among the three subgroups. For normally distributed data, a general linear model analysis of repeated measures was adopted to compare the three subgroups. Intergroup comparisons were performed using a paired t-test with a Bonferroni post-hoc test. Moreover, a linear single regression test was performed to detect the relationships between the morphological variables of the maxillary sinus and the cephalometric measurement variable for each group, including pretreatment and posttreatment. Multiple regression analysis, including the morphological variables regardless of the subgroup, was conducted to elucidate the correlations of the morphological measurement items as response variables to the cephalometric measurement items as explanatory variables. However, we included the factor of the classified skeletal group in this analysis as a dummy variable using forced entry, and appropriate cephalometric variables were extracted using a stepwise selection method. Probabilities below 0.05 as type I errors (α) were determined statistically significant.

## Results

Participants

The participants comprised 15 females with skeletal class I group, with an age range of 19 to 29 years (mean age ± SD, 24.5 ± 3.7 years), 15 females with skeletal class II group, with an age range of 18 to 28 years (23.4 ± 3.6 years), and 15 females with skeletal class III group, with an age range of 18 to 26 years (23.8 ± 3.1 years). There were no significant differences in age among the three subgroups (p = 0.704, one-way ANOVA). The treatment duration was 3.7 ± 1.1 years.

Volumetric and geometric measurements of the maxillary sinus

For all participants, no significant differences in the maxillary sinus dimensions were found between the left and right sides (p > 0.145), and the average values of the bilateral maxillary sinuses were adopted (Table 1). The pretreatment sinus width, height, and length in skeletal class I were 32.2 ± 3.9 mm (mean ± SD), 39.5 ± 3.8 mm, and 38.6 ± 2.4 mm, respectively. In skeletal class II, the sinus width, height, and length were 33.9 ± 6.2 mm, 37.3 ± 3.5 mm, and 38.6 ± 2.4 mm, respectively. In skeletal class III, width, height, and length were 32.0 ± 4.3 mm, 41.8 ± 5.0 mm, and 38.0 ± 2.8 mm, respectively. The total volumes of the left and right maxillary sinus were 36,179.3 ± 5,454.0 mm^3^ in the skeletal class I, 34,729.8 ± 6,686.6 mm^3^ in the skeletal class II, and 35,592.3 ± 10,334.3 mm^3^ in the skeletal class III. The values for width, length, and volume of the sinuses were almost similar among the three groups (p > 0.508); however, the skeletal class II group had a significantly lower height of the maxillary sinus compared to the skeletal class III group (p = 0.017).

**Table 1:** CT parameters of the maxillary sinus size for classified skeletal type Unit: mm for the width, height, and depth; mm^3^ for the volume.

	Class	Pretreatment	Posttreatment	p-value		
				Interaction	Time	Class
Width	Class I	32.2 ± 3.9	32.1 ± 3.9	0.344	0.722	0.508
	Class II	33.9 ± 6.2	33.9 ± 6.2			
	Class III	32.0 ± 4.3	32.0 ± 4.1			
Height	Class I	39.5 ± 3.8	39.6 ± 3.7	0.099	<0.001	0.020
	Class II	37.3 ± 3.5	37.8 ± 3.5			
	Class III	41.8 ± 5.0	42.2 ± 5.1			
Length	Class I	38.6 ± 1.8	38.7 ± 1.8	0.088	0.231	0.771
	Class II	38.6 ± 2.4	38.5 ± 2.5			
	Class III	38.0 ± 2.8	38.2 ± 2.8			
Volume	Class I	36,179.3 ± 5,454.0	36,716.7 ± 5,424.2	0.481	<0.001	0.858
	Class II	34,729.8 ± 6,686.6	35,136.2 ± 6,825.4			
	Class III	35,592.3 ± 10,334.3	36,414.6 ± 9,983.0			

Comparing the pretreatment and posttreatment measurements, the sinus width and length showed no significant changes during orthodontic treatment regardless of the skeletal pattern, whereas the posttreatment sinus height and volume were significantly greater than the pretreatment values, regardless of the skeletal classification (p < 0.01).

The relationship between craniofacial morphology and maxillary sinus morphology

The correlations between cephalometric measurements and maxillary sinus dimensions were analyzed (Table 2). In the skeletal class I group, the anteroposterior length of the anterior cranial base (SN) had a significant positive correlation with the maxillary sinus volume before and after treatment (p < 0.01). Furthermore, the pretreatment maxillary sinus volume showed significant positive correlations with SNA and ANB angles (p < 0.05). The posttreatment maxillary sinus width also had significant positive correlations with ANB angle, interincisal angle, and overjet (p < 0.05 or p < 0.01).

**Table 2:** Standardized coefficients between the measurement values for the maxillary sinus in relation to the cephalometric measurement variables analyzed by single linear regression analysis *p < 0.05; **p < 0.01.

(a) Skeletal class I jaw-base relationship																																																														
Pretreatment	SNA	SNB	ANB	Go. A	FMA		Occl. pl. A				Pal. pl. A				U1 to SN					IIA		IMPA			FMIA				SN				U1 to NA				L1 to NB				Overjet					Overbite		N-Me				Ar-Go				Ar-Me				Go-Me		
Width	0.426	0.433	0.202	0.176	0.121		-0.556				0.237				0.389					-0.256		-0.020			-0.094				0.331				0.338				0.204				0.405					0.200		0.058				-0.129				-0.108				-0.025		
Height	-0.010	-0.147	0.448	-0.022	-0.279		0.170				0.150				-0.231					0.237		0.126			0.142				0.405				-0.317				-0.197				-0.110					0.111		-0.430				0.321				0.242				-0.164		
Length	0.348	0.265	0.453	0.062	-0.076		-0.457				0.338				0.350					-0.183		-0.054			0.129				0.498				0.171				0.016				0.397					0.199		-0.276				0.339				0.420				-0.091		
Volume	0.543*	0.443	0.624*	0.058	-0.143		-0.469				0.311				0.413					0.277		0.199			-0.063				0.676**				0.236				0.111				0.491					0.184		-0.337				0.323				0.286				-0.191		
Posttreatment	SNA	SNB	ANB	Go. A	FMA		Occl. pl. A				Pal. pl. A				U1 to SN					IIA		IMPA			FMIA				SN				U1 to NA				L1 to NB				Overjet					Overbite		N-Me				Ar-Go				Ar-Me				Go-Me		
Width	0.455	0.308	0.652**	0.412	0.182		0.094				0.180				-0.318					0.591*		-0.403			0.385				0.334				-0.532				-0.382				0.521*					0.170		0.103				-0.114				-0.098				-0.039		
Height	-0.245	-0.210	-0.182	-0.151	-0.435		-0.177				-0.153				0.225					-0.328		0.333			-0.142				0.412				0.054				-0.006				-0.286					0.231		-0.507				0.301				0.256				-0.129		
Length	0.047	0.025	0.093	0.245	-0.239		-0.119				0.069				0.126					0.131		-0.118			0.294				0.460				-0.228				-0.352				0.175					0.120		-0.307				0.332				0.414				-0.073		
Volume	0.253	0.184	0.312	0.315	-0.164		-0.174				-0.003				0.036					0.286		-0.203			0.352				0.653**				-0.410				-0.455				0.295					0.342		-0.341				0.293				0.282				-0.217		
(b) Skeletal class II jaw-base relationship																																																														
Pretreatment	SNA	SNB	ANB	Go. A	FMA		Occl. pl. A				Pal. pl. A				U1 to SN					IIA		IMPA			FMIA				SN				U1 to NA				L1 to NB				Overjet					Overbite		N-Me				Ar-Go				Ar-Me				Go-Me		
Width	0.183	0.176	-0.009	-0.208	-0.114		-0.302				0.254				0.300					-0.032		-0.082			0.160				0.133				0.087				-0.127				0.236					0.403		0.226				0.170				0.170				0.171		
Height	-0.494	-0.318	-0.460	0.089	0.200		0.206				-0.249				-0.193					0.122		-0.442			0.167				-0.173				0.277				-0.337				0.275					0.091		0.524*				-0.293				-0.139				0.046		
Length	0.278	0.231	0.096	0.089	-0.098		-0.404				0.146				0.277					-0.031		-0.075			0.139				-0.100				0.098				0.057				0.122					0.322		-0.063				0.369				0.164				-0.178		
Volume	0.072	0.107	-0.122	-0.163	-0.090		-0.325				0.126				0.162					0.122		-0.311			0.314				-0.040				0.166				-0.288				0.381					0.516*		0.355				0.142				0.125				0.107		
Posttreatment	SNA	SNB	ANB	Go. A	FMA		Occl. pl. A				Pal. pl. A				U1 to SN					IIA		IMPA			FMIA				SN				U1 to NA				L1 to NB				Overjet					Overbite		N-Me				Ar-Go				Ar-Me				Go-Me		
Width	0.115	0.006	0.371	-0.189	-0.115		-0.102				0.041				0.008					0.050		0.105			0.013				0.113				-0.402				-0.085				0.511					0.402		0.257				0.151				0.149				0.154		
Height	-0.425	-0.369	-0.129	0.024	0.249		0.501				-0.164				-0.471					0.103		0.028			-0.286				-0.172				0.023				0.156				-0.305					-0.323		0.552*				-0.261				-0.111				0.082		
Length	0.271	0.150	0.385	0.080	-0.174		-0.097				-0.281				0.048					0.007		0.100			0.081				-0.104				-0.265				0.020				0.533*					0.302		-0.165				0.368				0.172				-0.177		
Volume	0.018	-0.047	0.233	-0.161	-0.134		0.082				-0.202				-0.120					-0.035		0.268			-0.130				-0.075				-0.245				0.024				0.378					0.139		0.314				0.150				0.124				0.129		
(c) Skeletal class III jaw-base relationship																																																														
Pretreatment	SNA	SNB	ANB	Go. A	FMA		Occl. pl. A				Pal. pl. A				U1 to SN					IIA		IMPA			FMIA				SN				U1 to NA				L1 to NB				Overjet					Overbite		N-Me				Ar-Go				Ar-Me				Go-Me		
Width	0.194	0.177	-0.008	0.127	0.194		-0.197				-0.319				-0.060					0.102		-0.065			-0.035				0.352				-0.107				0.045				-0.145					0.317		0.353				-0.077				0.684**				0.579*		
Height	0.095	0.083	0.019	0.157	0.145		-0.147				-0.199				-0.212					0.361		-0.271			0.287				0.221				-0.351				-0.180				-0.197					0.296		0.205				-0.003				0.590*				0.456		
Length	0.174	0.148	0.052	0.142	-0.013		-0.343				-0.220				-0.001					0.085		-0.018			0.032				0.518*				-0.217				-0.082				-0.139					0.340		0.058				0.279				0.507				0.325		
Volume	0.059	0.017	0.143	-0.025	0.048		-0.122				-0.323				-0.226					0.246		-0.091			0.096				0.289				-0.389				-0.088				-0.248					0.368		0.125				0.079				0.493				0.346		
Posttreatment	SNA	SNB	ANB	Go. A	FMA		Occl. pl. A				Pal. pl. A				U1 to SN					IIA		IMPA			FMIA				SN				U1 to NA				L1 to NB				Overjet					Overbite		N-Me				Ar-Go				Ar-Me				Go-Me		
Width	0.344	0.342	0.005	0.373	0.268		-0.610				-0.199				0.251					-0.043		-0.242			0.120				0.299				-0.079				-0.041				-0.180					0.167		0.262				-0.112				0.591*				0.533*		
Height	0.329	0.298	0.141	0.372	0.192		-0.506				0.002				0.311					0.010		-0.363			0.346				0.140				-0.153				-0.146				0.043					-0.095		0.110				-0.067				0.518*				0.442		
Length	0.386	0.318	0.289	0.148	-0.055		-0.594				-0.253				0.323					-0.166		0.047			-0.024				0.497				-0.164				0.020				-0.164					-0.061		-0.063				0.198				0.393				0.270		
Volume	0.246	0.179	0.279	0.183	0.075		-0.484				-0.175				0.298					-0.139		-0.080			0.048				0.260				-0.161				-0.040				-0.046					-0.060		-0.013				0.009				0.389				0.295		

For the skeletal class II group, the anterior facial height (N-Me) had a significant positive correlation with the maxillary sinus height before and after treatment (p < 0.05). Significant positive correlations were also found between the pretreatment overbite and the maxillary sinus volume and between the posttreatment overjet and the maxillary sinus length (p < 0.05).

In the skeletal class III group, total mandibular length (Ar-Me) and mandibular body length (Go-Me) were significantly positively correlated with the width of the maxillary sinus before and after treatment (p < 0.05 or p < 0.01). Furthermore, the pretreatment and posttreatment maxillary sinus heights showed a significant positive correlation with total mandibular length (p < 0.05). The posttreatment maxillary sinus width and length also had a significant negative correlation with the occlusal plane angle (p < 0.05).

Multiple regression analysis was used to analyze the correlations of maxillary sinus dimensions with 20 cephalometric variables, and multiple regression equations were calculated (Table 3). The effectiveness of each multiple regression equation was determined based on the probability level of the F-value. Pretreatment, the width, height, and length of the maxillary sinus were significantly related (probability level of F-value = 0.030, 0.005, and 0.045, respectively). In particular, overbite and U1-SN were significantly related to the maxillary sinus width (p = 0.007 and p = 0.044, respectively), which are regarded as response variables. Moreover, Ar-Me and SNB significantly affected maxillary sinus height (p = 0.010 and p = 0.041, respectively). Posttreatment, the maxillary sinus width and height were significantly improved (probability level of F-value = 0.016 and 0.016, respectively). In the multiple regression equation of maxillary sinus width, U1-NA and overjet were significantly affected (p = 0.006 and p = 0.009, respectively).

**Table 3:** Multiple regression analysis for the association of maxillary sinus dimensions with cephalometric parameters before and after orthodontic treatment Prob. F: probability level of F-value; R^2^: coefficient of determination. *p < 0.05; **p < 0.01.

1. Pretreatment	Model summary	
Multiple regression equation	Prob. F	R^2^
Width = 2.201 x (Class II) + 0.67 x (Class III) + 0.852** x (Overbite) + 0.158* x (U1-SN) + 12.705	0.030	0.230
Height = - 2.851 x (Class II) + 0.437 x (Class III) + 0.410* x (Ar-Me) - 0.425* x (SNB) + 28.531	0.005	0.305
Length = 0.413 x (Class II) - 1.808 x (Class III) - 0.168 x (Occ Plane to SN) + 0.12 x (Ar-Me) + 28.925**	0.045	0.211
Volume = - 683.899 x (Class II) + 1,724.558 x (Class III) + 1,102.423* x (Overbite) + 624.838 x (S-N) - 10643.691	0.068	0.192

2. Posttreatment	Model summary	
Multiple regression equation	Prob. F	R_^2^_
Width = 0.001 x (Class II) + 3.988 x (Class III) - 1.005** x (U1-NA) + 2.909** x (Overjet) + 26.967**	0.016	0.258
Height = - 1.715 x (Class II) + 0.573 x (Class III) + 0.217 x (Ar-Me) + 16.555	0.016	0.221
Length = - 0.167 x (Class II) + 0.281 x (Class III) - 0.195* x (Occ Plane to SN) - 0.308 x (U1-NA) + 43.856**	0.094	0.176
Volume = - 1,292.379 x (Class II) - 4,235.445 x (Class III) + 432.232 x (Ar-Me) - 9,171.899	0.307	0.083

## Discussion

A volumetric expansion of the maxillary sinus reduces skull weight and absorbs traumatic impacts on cranial skull structures [8]. However, the critical functions of the maxillary sinuses remain unclear. Because morphology reflects function, we hypothesized that maxillary sinus size is related to maxillomandibular morphology and studied the correlation of maxillary sinus dimensions to anteroposterior skeletal patterns. The present results indicated that the skeletal class II group had a significantly lower height of the maxillary sinus than the remaining two groups, whereas no significant differences in the sinus width, length, or volume were found among the three subgroups. Endo et al. [18] investigated the maxillary sinus dimension in different malocclusion types using two-dimensional lateral cephalograms and did not observe a significant correlation between the maxillary sinus size and the anteroposterior skeletal pattern. Oktay [19] also investigated the maxillary sinus size using orthopantomography and concluded that female participants with angle class II malocclusion had larger maxillary sinuses than the remaining groups. However, these studies showed two-dimensional data of the maxillary sinus based on a simple radiograph, and less information is available about the maxillary sinus dimensions. Abate et al. [20] first assessed the relationship of the maxillary sinus sizes and volume to a patient’s craniofacial characteristics using three-dimensional CT images and indicated that skeletal class II and III patients showed significantly smaller width of the maxillary sinus than skeletal class I patients, while no significant differences in the volume, height, and length of the maxillary sinus were found among the three skeletal classes. Furthermore, Shrestha et al. [21] evaluated the maxillary sinus volume in different skeletal patterns using CBCT and reported that the maxillary sinus volume was significantly larger in skeletal class II than in class III. These results are not consistent with our results; however, they have a common indication that the sagittal skeletal jaw-base relationship may affect the maxillary sinus dimensions and volume.

Previously, we studied the correlation between paranasal sinus and craniofacial morphology using CT data [22-24]. The frontal sinuses show increases in size and volume after orthodontic treatment during pubertal growth, whereas orthodontic treatment did not facilitate the postgrowth development in the frontal sinus [22,23]. This implies that the biomechanical stimulation caused by multibracket treatment may affect the development of frontal sinuses during the growth period, suggesting the potential contribution of early orthodontic treatment to frontal sinus development. In this study, the volume and dimensions of the maxillary sinus were longitudinally analyzed before and after orthodontic treatment. Regardless of the horizontal skeletal pattern, the height and volume of the maxillary sinus were significantly greater after treatment. This implies that biomechanical stimulation involving orthodontic treatment can facilitate the expansion of the maxillary sinus. However, our participants did not receive the same treatment procedure: for example, among 45 patients, 17 patients underwent posterior teeth distalization, and 28 patients did not receive any teeth distalization. Fifteen patients were treated with a lingual bracket appliance, but 30 patients were treated with a labial multibracket appliance. Furthermore, 39 patients underwent premolar extraction, but the remaining six patients did not receive premolar extraction. We could not find the significant effects of the premolar extraction, posterior teeth distalization, and type of multibracket appliance on the maxillary sinus dimensions. Ciğerim et al. [7] evaluated the influence of maxillary molar distalization on maxillary sinus volume and suggested that patients treated with molar distalization showed a significant increase in the maxillary sinus volume compared to patients without molar distalization. Portes et al. [16] evaluated the volumetric change of the maxillary sinus in patients who underwent miniscrew-anchored maxillary posterior tooth intrusion and suggested that miniscrew-anchored maxillary posterior en masse intrusion did not significantly affect the maxillary sinus volume. We divided our patients into two groups according to the orthodontic procedure: lingual bracket group and labial bracket group; molar distalization group and non-distalization group; or premolar extraction group and non-extraction group. However, we did not find any significant differences in the changes in maxillary sinus volume or dimensions during orthodontic treatment between the two groups. Further research with a larger sample size is needed to determine the influence of orthodontic procedures on maxillary sinus volume and dimensions.

Recently, the relationship of maxillary sinus dimensions to craniofacial morphology was assessed using CBCT [25-27]. However, most researchers have focused on the ANB angle or mandibular plane angle and evaluated the effects of the mandibular plane angle and/or the ANB angle on maxillary sinus dimensions and volume. In the present study, we investigated the relationship of maxillary sinus dimensions to 20 cephalometric parameters, including skeletal and denture parameters, using multiple regression analysis. The pretreatment U1-SN and overbite showed a significant correlation to the maxillary sinus width, and the posttreatment U1-NA and overjet also showed a significant correlation to the maxillary sinus width. This implies that anterior occlusion between the maxillary and mandibular incisors may affect the maxillary sinus width, suggesting that the maxillary sinus width may be a promising parameter for evaluating adequate anterior occlusion. Future studies should be performed to clarify treatment modalities and perform more detailed statistical analyses using a larger number of participants.

## Conclusions

We reported the average maxillary sinus dimensions and volume for postgrowth women and suggested that the values for width, length, and volume of the maxillary sinuses were almost similar irrespective of the skeletal classification, while the maxillary sinus height was significantly lower in the skeletal class II. Comparing the pretreatment and posttreatment measurements, the posttreatment sinus height and volume were significantly greater than the pretreatment values, although the sinus width and length showed no significant changes during orthodontic treatment. This implies that orthodontic treatment may facilitate the enlargement of the maxillary sinus even after physical growth. Furthermore, the maxillary sinus dimensions may be associated with craniofacial skeletal patterns and anterior occlusion. Future studies should be performed for more detailed statistical analyses using a larger number of participants.
